# Membrane-Induced Dichotomous Conformation of Amyloid β with the Disordered N-Terminal Segment Followed by the Stable C-Terminal β Structure

**DOI:** 10.1371/journal.pone.0146405

**Published:** 2016-01-05

**Authors:** Maho Yagi-Utsumi, Koichi Kato, Katsuyuki Nishimura

**Affiliations:** 1 Okazaki Institute for Integrative Bioscience, National Institutes of Natural Sciences, Okazaki, 444–8787, Japan; 2 Graduate School of Pharmaceutical Sciences, Nagoya City University, Nagoya, 467–8603, Japan; 3 Institute for Molecular Science, National Institutes of Natural Sciences, Okazaki, 444–8585, Japan; University of Pittsburgh School of Medicine, UNITED STATES

## Abstract

Various neurodegenerative disorders are ascribed to pathogenic molecular processes involving conformational transitions of amyloidogenic proteins into toxic aggregates characterized by their β structures. Accumulating evidence indicates that neuronal cell membranes provide platforms for such conformational transitions of pathogenic proteins as best exemplified by amyloid β (Aβ). Therefore, membrane-bound Aβ species can be promising targets for the development of novel drugs for Alzheimer’s disease. In the present study, solid-state nuclear magnetic resonance spectroscopy has elucidated the membrane-induced conformation of Aβ, in which the disordered N-terminal segment is followed by the stable C-terminal β strand. The data provides an insight into the molecular processes of the conformational transition of Aβ coupled with its assembly into parallel β structures.

## Introduction

Various neurodegenerative disorders are ascribed to pathogenic molecular processes involving conformational transitions of amyloidogenic proteins into toxic aggregates characterized by their β structures [1,2]. Amyloid β (Aβ) is a major player in the onset and development of Alzheimer’s disease (AD) [3]. The major isoform of this protein is composed of 40 or 42 amino acid residues, which is proteolytically cleaved from its precursor membrane protein. Aβ has a high propensity to form cross-β-fibrils [4–11], which are supposed to be a major component of senile plaque as a hallmark of AD. Accumulating evidence indicates that neuronal cell membranes provide platforms for conformational transition and subsequent aggregation of pathogenic proteins, including Aβ [12]. In particular, the formation of toxic Aβ aggregates is facilitated in membrane environments containing GM1 ganglioside, a glycosphingolipid abundant in neuronal cell membranes [13–15]. Therefore, membrane-bound Aβ species can be promising targets for the development of novel anti-AD drugs.

To characterize the membrane-bound conformation of Aβ, spectroscopic approaches, including solution nuclear magnetic resonance (NMR), circular dichroism (CD), and Fourier transform infrared spectroscopy (FT-IR), have been attempted by employing membrane mimics typified by aqueous micelles and even organic solvents [16–20]. The data thus obtained have underscored the formation of α-helical structures of Aβ in membrane-like environments. However, it has been suggested that the Aβ conformation is influenced by the size of micelles and the Aβ density thereon [21]. For example, Aβ has been reported to exhibit a thioflavin T-reactive β structure under conditions where Aβ density on micelles is high [21], although detailed structural information remains unavailable. These data emphasize the necessity of the conformational characterization of Aβ bound to large vesicles as more realistic model membranes. However, the membrane-bound Aβ molecules are not accessible with solution NMR techniques because of its slower molecular tumbling. In contrast, solid-state NMR studies have elucidated various structures of Aβ fibrils formed in solution [5,6,8,9,11] and membrane-associated Aβ fibrils [10,22–24], and also membrane perturbations caused by interactions of Aβ with lipid bilayers [25–28]. However, few detailed structural data have been available for the membrane-bound Aβ molecules prior to formation of amyloid fibrils.

Hence, in this study, we applied solid-state NMR spectroscopy to characterize the conformation of Aβ(1–40) bound to multilamella vesicles (MLVs) composed of 1,2-dimyristoyl-sn-glycero-3-phosphocholine (DMPC).

## Results and Discussion

**Fig 1:**
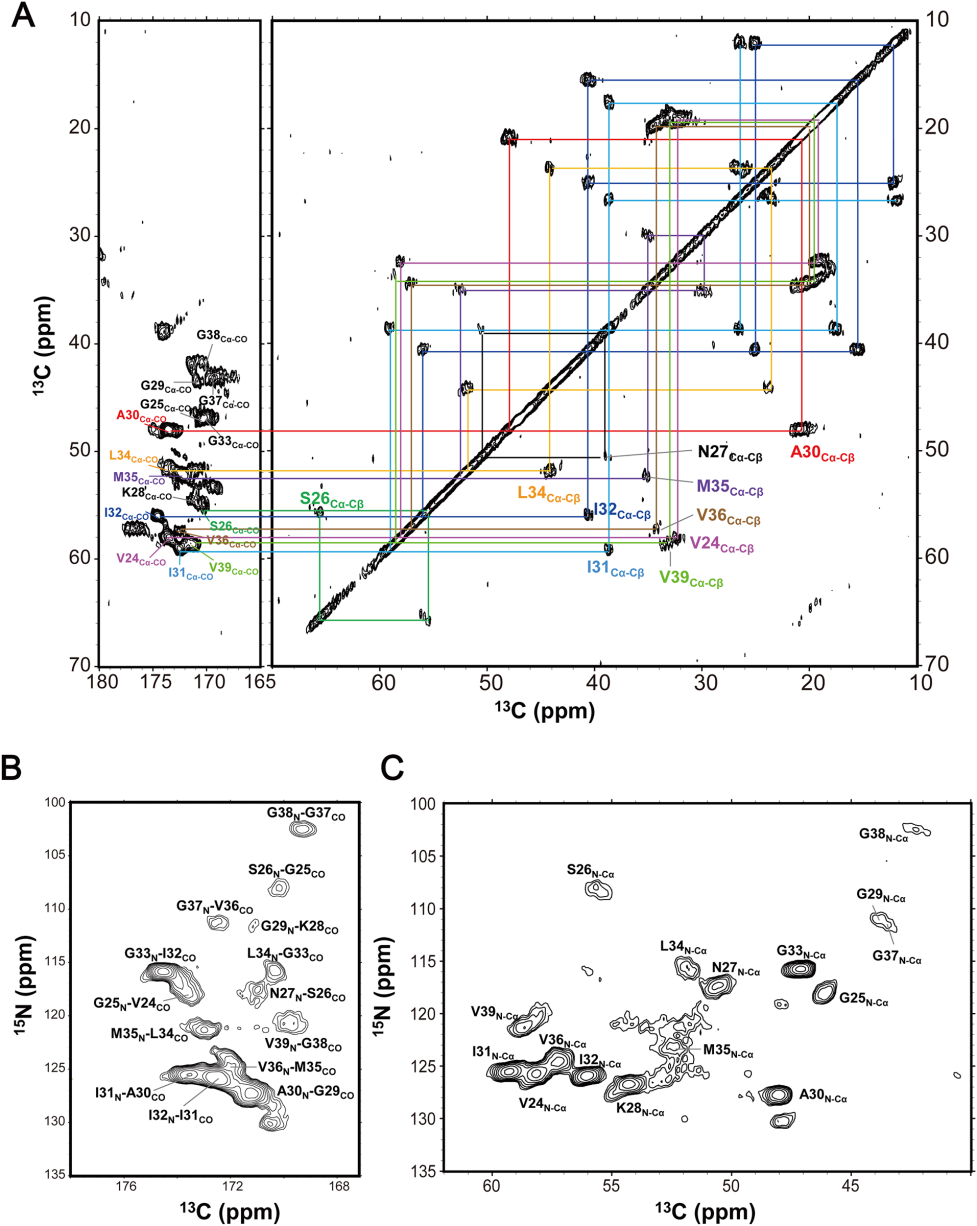
Solid-state NMR correlation spectra of [U-^13^C, ^15^N] Aβ(1–40) bound to DMPC bilayers for signal assignments. (A) ^13^C-CTUC-COSY homonuclear correlation spectrum. (B) NCO and (C) NCA heteronuclear correlation spectra based on DCP. All measurements were performed at 20°C for the sample (DMPC/Aβ(1–40) molar ratio = 10/1) in lyophilized and dry state.

Fig 1A shows the spectrum of uniformly ^13^C- and ^15^N-labeled Aβ(1–40) bound to DMPC lipid bilayers in lyophilized and dry state obtained by the ^13^C-constant time universal cross peak-COSY (CTUC-COSY) [29] homonuclear correlation experiment at 20°C. We observed 22 cross peaks, indicating that the Aβ(1–40) molecule bound to the DMPC membrane has a heterogeneous nature in terms of conformational variability. To identify the well-ordered region(s) of the membrane-induced Aβ(1–40) molecule, we attempted to assign the observed cross peaks based on through-bond connectivities. Sequential signal assignments were achieved by the ^13^C-CTUC-COSY homonuclear correlation experiment in conjunction with ^13^C observed NCO and NCA heteronuclear correlation experiments using ^13^C-^15^N double cross polarization (DCP) [30] as shown in Fig 1B and 1C. The peak assignments of Aβ(1–40) bound to DMPC lipid bilayers are summarized in Table 1. The results indicate that the observed peaks originated exclusively from the C-terminal segment of Aβ(1–40), i.e., Val24–Val39. The conformational-dependent isotropic chemical shift values of the peaks thus assigned were inspected using *TALOS-N* [31], revealing that the C-terminal segment exhibited a β structure (S1 Fig). In general, the β strands of Aβ peptides are stabilized through either intra- or intermolecular hydrogen-bonding interactions [4–11,14,32–36]. For example, it has been suggested that SDS can induce Aβ(1–42) oligomerization forming β-strands, Val18-Asp23 and Lys28-Gly33, which are arranged into strand-turn-strand structure [35]. Furthermore, an Aβ(1–40) oligomer induced by epigallocatechin-3-gallate adopts an intramolecular anti-parallel β-sheet formed between Glu22-Asp23 and Gly29-Val39 [36]. However, β-strand conformation has been continuously observed for the Gly25–Val39 segment as mentioned in present study (S1 Fig). Thus, the possibility of an anti-parallel β-sheet structure through intramolecular hydrogen bonds at this C-terminal regions was excluded. Moreover, previous ^2^H and ^31^P NMR data have demonstrated that Aβ(1–40) was not inserted in neutral lipid bilayers [26,27]. Based on these data, we concluded that the Aβ(1–40) molecules bound to DMPC lipid bilayers exhibit dichotomous conformation, in which the disordered N-terminal segment is followed by the stable C-terminal β structure.

**Table 1:** Summary of ^13^C, ^15^N isotropic chemical shifts with assignments of peaks for [U-^13^C, ^15^N] Aβ(1–40) incorporated into DMPC bilayers obtained by solid-state NMR.

Residue	Chemical shifts (ppm)					
	CO	Cα	Cβ	Cγ	Cδ	N
V24	173.6	58.1	32.4	19.7		125.7
G25	170.2	46.1				118.1
S26	171.0	54.8	65.6			107.9
N27	172.0	50.6	39.0			117.4
K28	171.2	54.3				126.7
G29	171.2	44.0				111.6
A30	173.5	48.0	20.7			127.7
I31	172.5	59.3	38.7	26.6, 17.5	12.0	125.5
I32	174.5	56.0	40.6	25.1, 15.5	12.1	125.9
G33	170.4	47.2				115.7
L34	173.0	51.8	44.2	23.9	26.8	115.8
M35	172.0	52.3	35.4	29.7		121.3
V36	172.6	57.2	34.4	21.3		124.6
G37	169.3	43.7				111.3
G38	170.0	42.3				102.5
V39	171.4	58.6	33.4	19.8		120.8
V40						

^13^C and ^15^N isotropic chemical shifts were referenced to ^1^H TMS by using secondary reference of adamantane and glycine for ^13^C and ^15^N, respectively.

**Fig 2:**
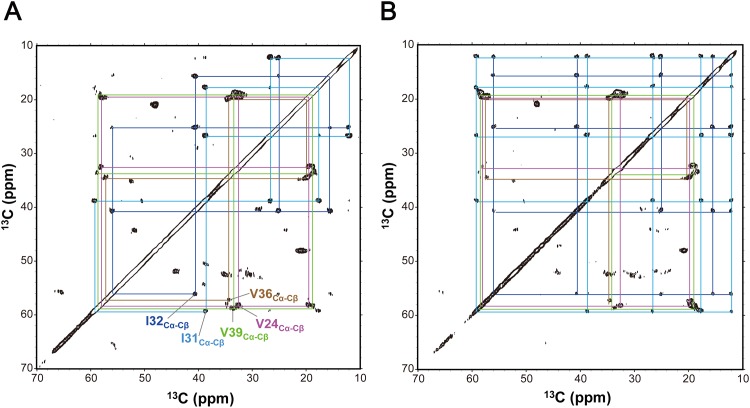
Solid-state NMR ^13^C homonuclear through-space correlation spectra of [U^−13^C, ^15^N] Aβ(1–40) bound to DMPC MLVs acquired by DARR/RAD with mixing times of (A) 10 ms and (B) 100 ms, respectively. All measurements were performed at 20°C for the sample (DMPC/Aβ(1–40) molar ratio = 10/1) in lyophilized and dry state.

The remaining possibilities of the β-strand structures due to intermolecular hydrogen bonding, i.e., parallel and/or anti-parallel β-sheet arrangements, were inspected using dipolar-assisted rotational resonance [37]/RF-assisted diffusion [38] (DARR/RAD) experiments. Huang *et*. *al*. indicated that, in 150-kDa oligomers of Aβ(1–42), its C-terminal β-strand is arranged into an intermolecular antiparallel β-sheet based on detection of inter-residue cross peaks in DARR spectra [32]. To clarify intermolecular proximity among Aβ(1–40) peptides in the DMPC-induced structure, we employed DARR/RAD experiments at various mixing times as shown in Fig 2 and S2 Fig. The ^13^C-homonuclear through-space DARR/RAD correlation spectra with mixing time up to 400 ms gave only intra-residue cross peaks and inter-residue cross peaks between adjacent residues as a hallmark of the anti-parallel β structure, suggesting that the C-terminal segment of Aβ(1–40) forms a parallel β structure in the membrane environment.

**Fig 3:**
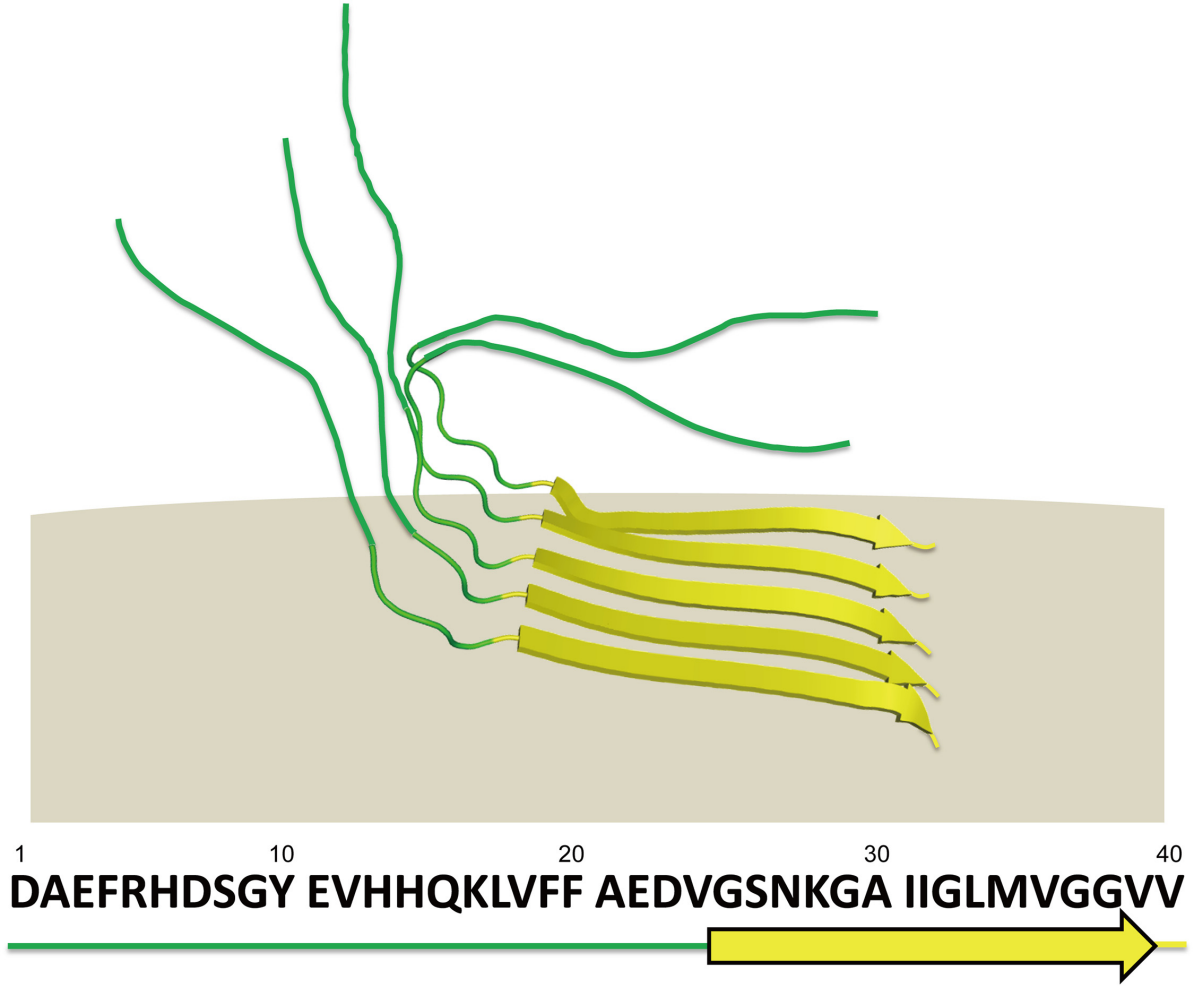
Structural model of Aβ(1–40) bound to DMPC bilayers characterized by solid-state NMR analyses together with amino acid sequence of Aβ(1–40).

The previously reported NMR studies have demonstrated the conformational versatility of the Aβ(1–40) segments. The free form of Aβ(1–40) is unstructured in an aqueous solution but exhibits an α-helical conformation at Gln15-Asp23 and Ile31-Met35 in organic solvents such as trifluoroethanol [18]. Upon binding to aqueous micelles composed of sodium dodecyl sulfate (SDS) or lyso-GM1, Aβ(1–40) forms discontinuous α-helices at His14-Val24 and Ile31-Val36 under micelle excess conditions [20]. In contrast, the C-terminal hydrophobic dipeptide segment shows two distinct conformational states that are reactive with thioflavin T in GM1 micelles under conditions where the Aβ density on the micelles is high [21]. In the typical mature cross-fibrils, Aβ(1–40) molecules adopt a strand-turn-strand motif at Tyr10-Val24 and Ala30-Val39 and assemble into parallel β sheets [8], although Aβ fibril exhibits polymorph because of the variety in the biological environments [10,39], not only because of the experimental conditions. Niu *et*. *al*. has recently reported solid-state NMR study for structure of Aβ(1–40) fibrils formed in the presence of lipid vesicles indicating significant structural differences from the fibrils formed in solution [24]. Furthermore, Qiang *et*. *al*. has recently reported solid-state NMR study characterizing the formation of a complex between membrane-associated Aβ(1–40) fibrils and lipids, which contains a typical β-loop-β motif similar to the mature fibril, suggesting that formation of such complex could be associated with lipid uptake and possibly membrane disruption [22]. On the other hand, CD and ^2^H and ^31^P NMR data have indicated that Aβ(1–40) could form either an α-helical structure or a β-structure depending on molar ratios of neutral/negatively charged lipids as well as the peptide/lipid molar ratios [26,27]. However, despite these cumulative observations, α-to-β conformational transition processes of Aβ(1–40) remain largely unknown at the atomic level. The present solid-state NMR study identified a partially ordered conformation of membrane-induced Aβ(1–40) molecules, in which only the C-terminal segments are involved in a parallel β structure, while leaving the N-terminal segment disordered (Fig 3). The stabilized C-terminal segment encompasses the C-terminal half of the strand-turn-strand structure in the amyloid fibrils. Based on all these data, we conclude that DMPC vesicles provide large, flat surfaces compared with those of micelles and thereby promote intermolecular interactions of Aβ(1–40) through their C-terminal segments, which form a stable core of the Aβ(1–40) assembly. We suggest that DMPC vesicles capture an intermediate form of Aβ(1–40) during its conformational transition coupled with assembly into the toxic β structure, which could be delineated by our solid-state NMR approach. Our findings will not only provide unique insights into the mechanisms underlying Aβ assembly but also offer structural clues for designing drugs targeting the assembly intermediates of Aβ for the development of anti-AD therapeutics.

## Materials and Methods

### Sample preparation

Expression and purification of uniformly ^13^C- and ^15^N-labeled Aβ(1–40) were performed as previously described [21]. DMPC was purchased from Avanti Polar Lipids, Inc. MLVs were prepared by dissolving 7.0 mg of DMPC in 100 μl of methanol/chloroform (1:1) solution and 3.5 mg of Aβ(1–40) peptide in 500 μl of chloroform (DMPC: Aβ(1–40) = 10:1). The solvents in the DMPC/Aβ(1–40) sample were removed from the sample by nitrogen gas, followed by complete drying under vacuum. The dried DMPC/Aβ(1–40) sample was suspended in a total of 400 μl of ultra-pure water and homogenized by six cycles of successive freezing, thawing, and vortexing for 5 min each. Then, the DMPC/Aβ(1–40) sample was immediately lyophilized to capture the structural state of Aβ(1–40) induced upon the interaction with DMPC bilayers.

### Solid-state NMR experiments

All solid-state NMR experiments were performed using a Bruker Avance 600 spectrometer at ^1^H resonant frequency of 600 MHz, equipped with a 2.5 mm outer diameter ^1^H-^13^C-^15^N triple resonance magic angle spinning (MAS) probe at a spinning frequency of 13.5 kHz. The sample was packed into a 4 mm space at the center of a sample tube using original Diflon spacers of 1 mm thickness to maintain RF homogeneity. Temperatures were controlled to 20°C using a VT controller. Typical RF fields of ^1^H and ^13^C pulses were 100 and 88 kHz, respectively. The spinning frequency of the sample was actively controlled by Bruker MAS controller to 13.5 kHz ± 5 Hz. ^1^H heteronuclear dipolar decoupling was achieved by SPINAL64 [40] at a RF field of 100 kHz. ^1^H-X RAMP-Cross polarizations [41] were used to enhance the initial magnetization of rare nuclei with ^1^H RF fields of 100 and 80 kHz for X = ^13^C, ^15^N, respectively with repetition time of 2 s.

The ^13^C CTUC-COSY experiment was acquired at a constant evolution time of 6.96 ms with *t*_1_ of 348 and *t*_2_ of 2500 points. The number of scans was 1024 for each *t*_1_ point. Z-filtering of 1 ms was inserted before detection.

The NCO and NCA-DCP experiments were acquired with *t*_1_ of 256 and *t*_2_ of 2048 points, respectively. The number of scans was 1024 for each *t*_1_ point. Contact times of 1.5 and 5.0 ms were used for ^1^H-^15^N and ^15^N-^13^C CPs, respectively. ^15^N-^13^C CP was achieved by a ^13^C spin locking field of 20 kHz at -1 SSB HH matching condition with 5% of RF ramp of ^15^N RF spin locking field. LG decoupling at RF field of 100 kHz was applied during the second CP period.

The ^13^C DARR/RAD experiments were performed at n = 1 R^3^ condition for ^1^H RF field during the mixing period with *t*_*1*_ of 500 and *t*_*2*_ of 2500 points, respectively. The number of scans was 256 for each *t*_*1*_ point. The experiments were performed at mixing times of 4, 10, 100, 200, and 400 ms.

Quadrature detection in the indirect dimensions was achieved by States procedure for all two-dimensional (2D) experiments. Other parameters for 2D experiments are described in individual figure legends. The *t*_*1*_ time domain of 2D FID for ^13^C CTUC-COSY experiment was extended by forward linear prediction. The *t*_*1*_ time domains of 2D FIDs were apodized by a sin window function. 2D FIDs were zero filled to 4096 for both *t*_*1*_ and *t*_*2*_ time domains prior to Fourier transformation. All data processing was performed using TOPSPIN2.1 (Bruker Biospin, Japan). Spectral analyses were performed using Sparky. Secondary structural elements were identified by *TALOS-N* software [31] according to ^13^C and ^15^N chemical shifts.

## Supporting Information

S1 Fig Dihedral angles (φ, ψ) obtained by *TALOS-N* analysis according to ^13^C and ^15^N chemical shifts of Aβ(1–40) bound to DMPC MLVs. The secondary structure analysis by the TALOS software indicated a β-strand region (yellow shadow and yellow arrows) at the residues of Gly25-Val39. The chemical shift references were corrected before analyses of *TALOS-N*.
(PDF)

S2 Fig Solid-state NMR ^13^C homonuclear through-space correlation spectra of [U-^13^C, ^15^N] Aβ(1–40) bound to DMPC MLVs acquired by DARR/RAD with mixing times of (A) 4 ms, (B) 200 ms, and (C) 400 ms, respectively. All measurements were performed at 20°C for the sample (DMPC/Aβ(1–40) molar ratio = 10/1) in lyophilized and dry state.
(PDF)
