# Supplementary material for: Membrane-Induced Dichotomous Conformation of Amyloid β with the Disordered N-Terminal Segment Followed by the Stable C-Terminal β Structure
Source: PLoS One. 2016 Jan 5;11(1):e0146405. doi: 10.1371/journal.pone.0146405 (PMC4701388; doi:10.1371/journal.pone.0146405)
Supplement: S1 Fig — The secondary structure analysis by the TALOS software indicated a β-strand region (yellow shadow and yellow arrows) at the residues of Gly25-Val39. The chemical shift references were corrected before analyses of TALOS-N. (PDF) [file pone.0146405.s001.pdf]

Figure S1

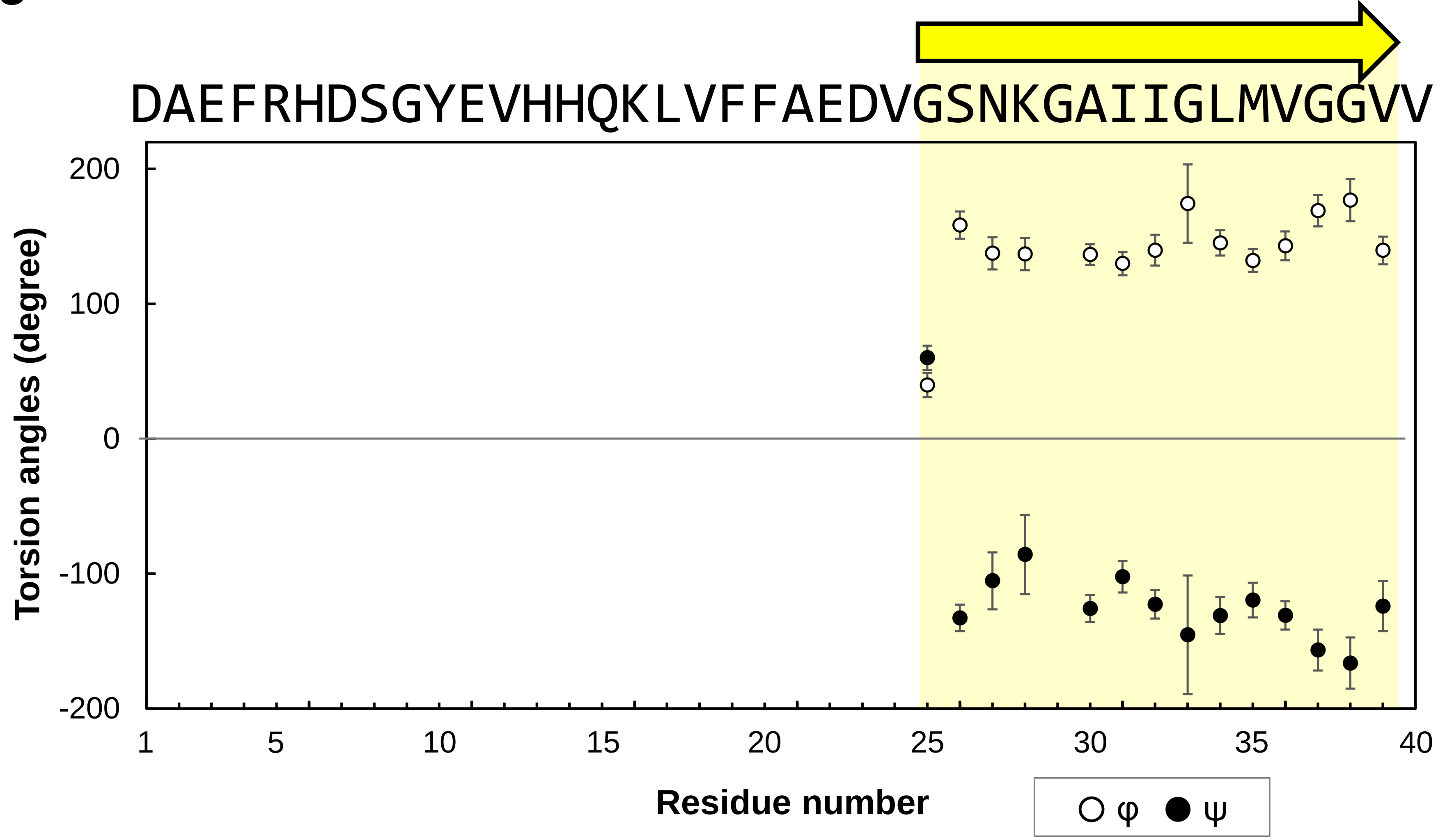

Figure S1

Dihedral angles (φ, ψ) obtained by *TALOS-N* analysis according to ^{13}C and ^{15}N chemical shifts of Aβ(1-40) bound to DMPC MLVs. The secondary structure analysis by the TALOS software indicated a β-strand region (yellow shadow and yellow arrows) at the residues of Gly25-Val39. The chemical shift references were corrected before analyses of *TALOS-N*.
