# Supplementary material for: Membrane-Induced Dichotomous Conformation of Amyloid β with the Disordered N-Terminal Segment Followed by the Stable C-Terminal β Structure
Source: PLoS One. 2016 Jan 5;11(1):e0146405. doi: 10.1371/journal.pone.0146405 (PMC4701388; doi:10.1371/journal.pone.0146405)
Supplement: S2 Fig — All measurements were performed at 20°C for the sample (DMPC/Aβ(1–40) molar ratio = 10/1) in lyophilized and dry state. (PDF) [file pone.0146405.s002.pdf]

Figure S2

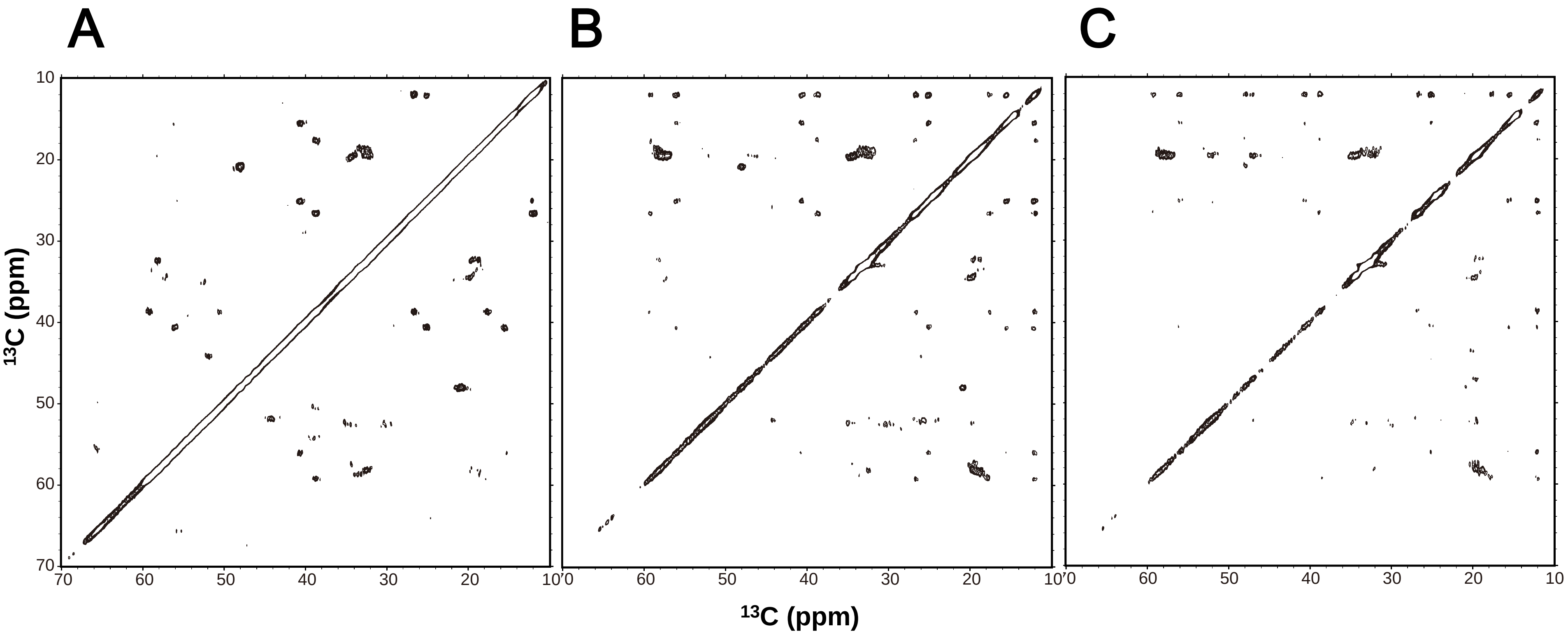

Figure S2
Solid-state NMR ^{13}C homonuclear through-space correlation spectra of [U-^{13}C, ^{15}N] Aβ(1-40) bound to DMPC MLVs acquired by DARR/RAD with mixing times of (A) 4 ms, (B) 200 ms, and (C) 400 ms, respectively. All measurements were performed at 20℃ for the sample (DMPC/Aβ(1-40) molar ratio = 10/1) in lyophilized and dry state.
